# Supplementary figures and images for: Timeliness and missed opportunities for vaccination among children aged 0 to 23 months in Dschang health district, West region, Cameroon: A cross-sectional survey
Source: PLOS Glob Public Health. 2023 Jun 14;3(6):e0001721. doi: 10.1371/journal.pgph.0001721 (PMC10266616; doi:10.1371/journal.pgph.0001721)

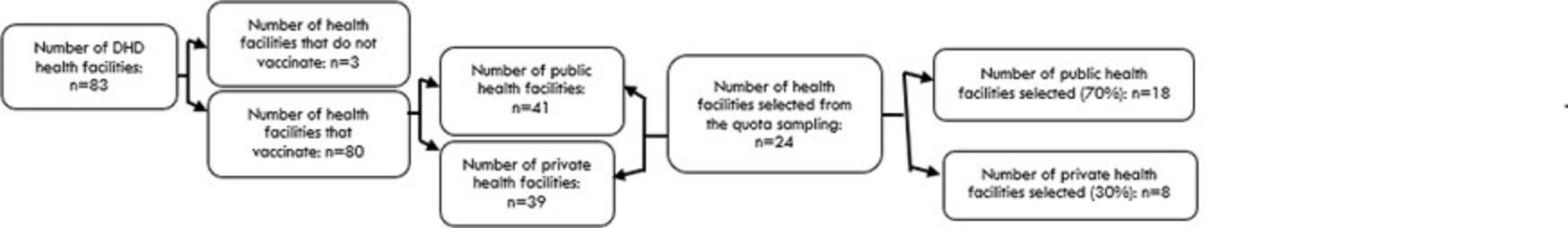

Supplement: S1 Fig — (TIF) [file pgph.0001721.s001.tif]
