# Supplementary material for: Timeliness and missed opportunities for vaccination among children aged 0 to 23 months in Dschang health district, West region, Cameroon: A cross-sectional survey
Source: PLOS Glob Public Health. 2023 Jun 14;3(6):e0001721. doi: 10.1371/journal.pgph.0001721 (PMC10266616; doi:10.1371/journal.pgph.0001721)
Supplement: S1 Table — (DOCX) [file pgph.0001721.s002.docx]

**S1 Table**: source of immunization among children aged 0-23 months in the DHD by doses of vaccines received in 2021.

| **Antigens** | Vaccination record + caregivers declarations (%) | Vaccination record + vaccination dates (%) | Vaccination record + tick mark (%) | Total doses take |
| --- | --- | --- | --- | --- |
| **BCG** | 4 (1.3) | 302 (98.86) | 0 | 306 |
| **HepB0** | 1 (1.45) | 68 (98.55) | 0 | 69 |
| **OPV0** | 6 (1.75) | 335 (97.95) | 1 (0.29) | 342 |
| **OPV1** | 6 (2.29) | 256 (97.7) | 0 | 262 |
| **Rota1** | 6 (2.29) | 256 (97.7) | 0 | 262 |
| **PCV1** | 5 (1.91) | 257 (98.1) | 0 | 262 |
| **Penta1** | 5 (1.92) | 255 (98.1) | 0 | 260 |
| **OPV2** | 7 (3.32) | 204 (96.68) | 0 | 211 |
| **Rota2** | 7 (3.33) | 203 (96.67) | 0 | 210 |
| **PCV2** | 5 (2.39) | 204 (96.61) | 0 | 209 |
| **Penta2** | 5 (2.38) | 205 (97.62) | 0 | 210 |
| **OPV3** | 3 (1.79) | 165 (98.21) | 0 (0.0) | 168 |
| **IPV** | 3 (1.80) | 164 (98.20) | 0 (0.0) | 167 |
| **PCV3** | 4 (2.38) | 164 (97.62) | 0 (0.0) | 168 |
| **Penta3** | 4 (2.38) | 164 (97.62) | 0 (0.0) | 168 |
| **MM1** | 0 (0.0) | 79 (100.0) | 0 (0.0) | 79 |
| **YF** | 0 (0.0) | 74 (100.0) | 0 (0.0) | 74 |
| **MM2** | 0 (0.0) | 11 (100.0) | 0 (0.0) | 11 |
| **Total** | 71 (2.06) | **3366 (97.79)** | 1 (0.02) | **3438** |

Abbreviations: **BCG**: Bacillus Calmette Guerin; **OPV**: oral polio vaccine; **Penta**: Diphtheria-tetanus-pertussis-hepatitis B Haemophilus influenzae type b; **PCV**: pneumococcal conjugated vaccine; **Rota**: rotavirus; **MR**: measles-rubella, **YF**: yellow fever.

Of the 363 children included in our analysis, 97.79% (3366/3438) of vaccine doses were correctly documented (presentation of the booklet with vaccination dates). The main source of vaccination was the vaccination record with at least one vaccination date for each vaccine received by the child.
