# Supplementary material for: Timeliness and missed opportunities for vaccination among children aged 0 to 23 months in Dschang health district, West region, Cameroon: A cross-sectional survey
Source: PLOS Glob Public Health. 2023 Jun 14;3(6):e0001721. doi: 10.1371/journal.pgph.0001721 (PMC10266616; doi:10.1371/journal.pgph.0001721)
Supplement: S2 Table — (DOCX) [file pgph.0001721.s003.docx]

**S2 Table**: Vaccination timeliness among children with cards seen and dates in the DHD in 2021

|  | **Absolute Frequency** | | | | **Proportion (%)** | | |
| --- | --- | --- | --- | --- | --- | --- | --- |
| **Vaccines** | **N*** | **Early** | **On_time** | **Delayed** | **Early_p** | **On_time_p** | **Delayed_p** |
| BCG | 240 | 0 | 105 | 135 | 0 | 43.75 | 56.25 |
| OPV0 | 278 | 0 | 117 | 161 | 0 | 42.09 | 57.91 |
| MCV1 | 5 | 2 | 1 | 2 | 40 | 20 | 40 |
| YF | 2 | 1 | 1 | 0 | 50 | 50 | 0 |
| ROTA1 | 196 | 31 | 151 | 14 | 15.82 | 77.04 | 7.14 |
| ROTA2 | 142 | 18 | 107 | 17 | 12.68 | 75.35 | 11.97 |
| PENTA1 | 193 | 30 | 149 | 14 | 15.54 | 77.2 | 7.25 |
| PENTA2 | 141 | 17 | 106 | 18 | 12.06 | 75.18 | 12.77 |
| PENTA3 | 98 | 9 | 73 | 16 | 9.18 | 74.49 | 16.33 |
| IPV | 98 | 10 | 73 | 15 | 10.2 | 74.49 | 15.31 |
| OPV1 | 197 | 35 | 149 | 13 | 17.77 | 75.63 | 6.6 |
| OPV2 | 143 | 18 | 108 | 17 | 12.59 | 75.52 | 11.89 |
| OPV3 | 98 | 9 | 74 | 15 | 9.18 | 75.51 | 15.31 |
| PCV1 | 196 | 32 | 150 | 14 | 16.33 | 76.53 | 7.14 |
| PCV2 | 141 | 17 | 107 | 17 | 12.06 | 75.89 | 12.06 |
| PCV3 | 99 | 9 | 74 | 16 | 9.09 | 74.75 | 16.16 |

*Number of children who received the vaccine on card
