# Supplementary material for: Timeliness and missed opportunities for vaccination among children aged 0 to 23 months in Dschang health district, West region, Cameroon: A cross-sectional survey
Source: PLOS Glob Public Health. 2023 Jun 14;3(6):e0001721. doi: 10.1371/journal.pgph.0001721 (PMC10266616; doi:10.1371/journal.pgph.0001721)
Supplement: S3 Table — (DOCX) [file pgph.0001721.s004.docx]

**S3 Table**: Socio demographic information of health workers from surveyed health facilities in the DHS in 2021

| **Modalities** | Effectives | Proportions (%) |
| --- | --- | --- |
| **Gender of health personnel** | | |
| Male | 22 | 25 (22/88) |
| Female | **66** | **75 (66/88)** |
| **Initial training of health personnel** | | |
| Nurse | **50** | **56.82 (50/88)** |
| Midwife | 7 | 7.95 (7/88) |
| Nursing assistant | 6 | 6.82 (6/88) |
| Laboratory technician | 17 | 19.32 (17/88) |
| General practitioner | 7 | 7.95 (7/88) |
| Pharmacy clerk | 1 | 1.14 (1/88) |
| **Age group of health personnel** | | |
| Less than 25 years old | 10 | 11.36 (10/88) |
| 25-35 years old | **51** | **57.95 (51/88)** |
| 36-45 years old | 14 | 15.91 (14/88) |
| Over 45 years of age | 13 | 14.77 (13/88) |
| **Service employed** | | |
| Preventive | **53** | **60.23 (53/88)** |
| Curative | 35 | 39.77 (35/88) |
| **Health facility category** | | |
| District Hospital | 22 | 25 (22/88) |
| District Medical Center | 9 | 10.23 (9/88) |
| Integrated Health Center | 28 | 31.82 (28/88) |
| Health Center | **29** | **32.95 (29/88)** |
| **Type de health facility** | | |
| Publique | **59** | **67.05 (59/88)** |
| Private | 29 | 32.95 (29/88) |
