# Supplementary material for: Timeliness and missed opportunities for vaccination among children aged 0 to 23 months in Dschang health district, West region, Cameroon: A cross-sectional survey
Source: PLOS Glob Public Health. 2023 Jun 14;3(6):e0001721. doi: 10.1371/journal.pgph.0001721 (PMC10266616; doi:10.1371/journal.pgph.0001721)
Supplement: S4 Table — (DOCX) [file pgph.0001721.s005.docx]

**S4 Table**: Attitudes of health personnel in the Dschang health district towards immunization in 2021

| Modalities | Numbers (N=88) | % | |
| --- | --- | --- | --- |
| **In which of the following situations should you ask for the doses that children received and the missing doses by age?** | | |  |
| During a routine visit to the child | **65** | **73.86** | |
| During a consultation for any illness | 62 | 70.45 | |
| When a child accompanies a parent to the health centre | 37 | 42.05 | |
| All of the above | 5 | 5.68 | |
| **Who is responsible for assessing children's immunization status** | | |  |
| The child's parents | **22** | **25** | |
| The health worker responsible for immunization | 44 | 78.41 | |
| Outpatient physicians. inpatient departments | **37** | **42.04** | |
| All of the above | 27 | 30.68 | |
| **What are the reasons why some children are not up to date with their immunizations? (n=85)** | | |  |
| Parents' negative beliefs about immunization | **65** | **76.47** | |
| Inconsistent immunization times with parents' schedules | **44** | **51.76** | |
| Nurses do not ask for children's immunization schedules | 31 | 36.47 | |
| Nurses do not review children's immunization records | 1 | 1.18 | |
| False contraindications to vaccination noted by health workers | 26 | 30.59 | |
| Long distance between the vaccination site and homes | **45** | **52.94** | |
| Vaccine stock-outs | 2 | 2.35 | |
