# Supplementary material for: Timeliness and missed opportunities for vaccination among children aged 0 to 23 months in Dschang health district, West region, Cameroon: A cross-sectional survey
Source: PLOS Glob Public Health. 2023 Jun 14;3(6):e0001721. doi: 10.1371/journal.pgph.0001721 (PMC10266616; doi:10.1371/journal.pgph.0001721)
Supplement: Attachment — Submitted filename: Reponses to reviewers.docx [file pgph.0001721.s008.docx]

Reponses to Reviewers

PGPH-D-22-01589
Missed opportunities for vaccination and associated factors among children aged 0 to 23 months in Dschang health district, West region, Cameroon: a cross-sectional survey
PLOS Global Public Health

**Comments to the Author**

1. Does this manuscript meet PLOS Global Public Health’s [publication criteria](https://journals.plos.org/globalpublichealth/s/criteria-for-publication)? Is the manuscript technically sound, and do the data support the conclusions? The manuscript must describe methodologically and ethically rigorous research with conclusions that are appropriately drawn based on the data presented.

Reviewer #1: Partly

Reviewer #2: Yes

2. Has the statistical analysis been performed appropriately and rigorously?

Reviewer #1: Yes

Reviewer #2: Yes

ANSWER/

We are grateful for these positive comments from the reviewers. To increase the consistent of the results, we cross-checked the analysis, and clarify some missed points in the text. We re-ran the timeliness analysis and present the figure in the main text. We also used the occasion to improve the MOV analysis method by following the rigorous World health Organization methodology.

3. Have the authors made all data underlying the findings in their manuscript fully available (please refer to the Data Availability Statement at the start of the manuscript PDF file)?

The [PLOS Data policy](https://journals.plos.org/globalpublichealth/s/data-availability) requires authors to make all data underlying the findings described in their manuscript fully available without restriction, with rare exception. The data should be provided as part of the manuscript or its supporting information, or deposited to a public repository. For example, in addition to summary statistics, the data points behind means, medians and variance measures should be available. If there are restrictions on publicly sharing data—e.g. participant privacy or use of data from a third party—those must be specified.

Reviewer #1: No

ANSWER: the main dataset analyzed is used by a third party, reason why it can not be shared with the main text. However, we have made available all additional information that complement the results to enable more understanding of these results, available to readers.

Reviewer #2: Yes

ANSWER

We are grateful, and thank the reviewer 2 for his comment that is aligned with our previous response to reviewer 1.

4. Is the manuscript presented in an intelligible fashion and written in standard English?

PLOS Global Public Health does not copyedit accepted manuscripts, so the language in submitted articles must be clear, correct, and unambiguous. Any typographical or grammatical errors should be corrected at revision, so please note any specific errors here.

Reviewer #1: Yes

Reviewer #2: Yes

ANSWER

We thank the editor for their comments. We used this opportunity to re-edit the manuscript and correct typo errors, and check for consistency within sentences. We do hope, the article is readable and understandable.

5. Review Comments to the Author

Please use the space provided to explain your answers to the questions above. You may also include additional comments for the author, including concerns about dual publication, research ethics, or publication ethics. (Please upload your review as an attachment if it exceeds 20,000 characters)

**#====================================**

**# SPECIFIC RESPONSES TO REVIEWER #1**

**#====================================**

Reviewer #1: Introduction
There are various typos in the introduction
"Indeed, it prevents nearly 75,000 children from suffering serious physical, mental or neurological disabilities [1]" this number doesn't seem correct . It is too low.
What is RR1 ?
The second last paragraph of introduction is more suited for discussion
**Answer: We thank the reviewer for the interesting comment and feedback. Of course, this seems too low regarding actual evidence: reason why the figure was deleted in the text.**

**RR1 stood for the first dose of measles administered at 9 months. This was replaced by MCV1, and defined in the text as the measles-rubella first dose.**

Methods
What is DHD?
what is meant by couples/children ? is there a better terminology that can be used ?
What is WHO quota sampling ?
What is CAP survey ?
**Answer: We thank the reviewer for the interesting comments that obviously drive our attention. The notation DHD stands for Dschang Health District. This notation has been revised and extended completely in the text. In some cases, we replaced it by “district” only.**

- **The term “couples/children” was replaced by the most suited expression. We meant children with their caregivers.**
- **“WHO quota sampling” refered to an existing literature that used the same sampling method recommended by the World Health Organization to estimate the number of health workers for the KAP study. To avoid misunderstanding, we simply reformulated that in the text, and mentioned that we set a quota of health workers, and divided that into the number of health facilities in the district.**
- **“CAP” study was a mistake; it stood for KAP; we have made the changes in the text, and provided the definition: knowledge, attitude/bahaviour and practice.**

Results

One decimal point should be enough
Please don't start sentences with numbers
Results are too long . some of the tables can be moved to appendix.
**Answer: We thank the reviewer for the interesting comments and apologize for all these insufficiencies. We went through the text and left numbers with one decimal point; sentences were revised as suggested, and we also put some tables as supplementary tables.**

Conclusion needs to be crisp and not more than 2-3 sentences

**Answer: We thank the reviewer for the remark. The conclusion has been shortened as suggested.**

**#====================================**

**# SPECIFIC RESPONSES TO REVIEWER #2**

**#====================================**

Reviewer #2: The manuscript numbering system was not done making review process difficult
Page 1
The title is topical as the EPI vaccination schedule in in developing countries and completion of RI is still a problem and the needs for more studies is appropriate

**ANSWER**

**We thank you for the comments, and added a little changes in the title.**

Abstract
....in the west region
MOV prevalence range from 69.7%(19/29) to 98.5%(330/335)
...74%asked parents
... strategies for remedying this includes
**Answer: All changes are done!**

Page 2
....remove expanded program on immunization after the ref
In Cameroon vaccination has been offered throughout the country
**Answer: We thank the reviewer for the attention made on the text. All changes are done!**

Table2: Types of vaccination...
Table 5: Sociodemographic information of health workers...
Table 9: Logistic regression of MOV among children...
**Answer: We thank the reviewer for the attention made on the text. All changes were made appropriately! After running the analysis, the logistic regression was no helpful, so the previous results were replaced by new ones, and we stated that more investigation is needed to explore factors that increase the risk of MOV.**

Discussion
Page 16
**Answer: We thank the reviewer pointing out this page for revision Indeed, we re-edited the text accordingly, and all changes are visible in the discussion.**

6. PLOS authors have the option to publish the peer review history of their article ([what does this mean?](https://journals.plos.org/globalpublichealth/s/editorial-and-peer-review-process#loc-peer-review-history)). If published, this will include your full peer review and any attached files.


**Do you want your identity to be public for this peer review?** If you choose “no”, your identity will remain anonymous but your review may still be made public.


For information about this choice, including consent withdrawal, please see our [Privacy Policy](https://www.plos.org/privacy-policy).

Reviewer #1: No

Reviewer #2: No

[NOTE: If reviewer comments were submitted as an attachment file, they will be attached to this email and accessible via the submission site. Please log into your account, locate the manuscript record, and check for the action link "View Attachments". If this link does not appear, there are no attachment files.]

While revising your submission, please upload your figure files to the Preflight Analysis and Conversion Engine (PACE) digital diagnostic tool, <https://pacev2.apexcovantage.com/>. PACE helps ensure that figures meet PLOS requirements. To use PACE, you must first register as a user. Registration is free. Then, login and navigate to the UPLOAD tab, where you will find detailed instructions on how to use the tool. If you encounter any issues or have any questions when using PACE, please email PLOS at [figures@plos.org](mailto:figures@plos.org). Please note that Supporting Information files do not need this step.
