# Supplementary material for: Timeliness and missed opportunities for vaccination among children aged 0 to 23 months in Dschang health district, West region, Cameroon: A cross-sectional survey
Source: PLOS Glob Public Health. 2023 Jun 14;3(6):e0001721. doi: 10.1371/journal.pgph.0001721 (PMC10266616; doi:10.1371/journal.pgph.0001721)
Supplement: Attachment — Submitted filename: Reponses to reviewers.docx [file pgph.0001721.s009.docx]

Responses to Editor’s comments

Journal Requirements:

1. Please amend your detailed Financial Disclosure statement. This is published with the article. It must therefore be completed in full sentences and contain the exact wording you wish to be published.
   a. Please clarify all sources of funding (financial or material support) for your study. List the grants (with grant number) or organizations (with url) that supported your study, including funding received from your institution. 
   b. State the initials, alongside each funding source, of each author to receive each grant.
   c. State what role the funders took in the study. If the funders had no role in your study, please state: “The funders had no role in study design, data collection and analysis, decision to publish, or preparation of the manuscript.”
   d. If any authors received a salary from any of your funders, please state which authors and which funders.
   If you did not receive any funding for this study, please simply state: “The authors received no specific funding for this work.”

ANSWER

This work was not funded; we state the following: “The authors received no specific funding for this work.” This sentence was also added in the funding section of the manuscript.

2. We do not publish any copyright or trademark symbols that usually accompany proprietary names, eg (R), (C), or TM  (e.g. next to drug or reagent names). Please remove all instances of trademark/copyright symbols throughout the text, including R on page 4 and TM on page 3.

ANSWER

We thank the Editor for this interesting remark. We have removed trademark symbols on pages 3 and 4.

3. We have noticed that you have uploaded Supporting Information files, but you have not included a list of legends. Please add a full list of legends for your Supporting Information files after the references list.

ANSWER

Dear Editor, thank you for coming back on the point. We uploaded a file previously as you mentioned it, without adding the legend in the text. In the current, we have listed supplementary file legend and uploaded the main file containing these materials.

4. In the online submission form, you indicated that [Insert text from online submission form here]. All PLOS journals now require all data underlying the findings described in their manuscript to be freely available to other researchers, either 1. In a public repository, 2. Within the manuscript itself, or 3. Uploaded as supplementary information.
   This policy applies to all data except where public deposition would breach compliance with the protocol approved by your research ethics board. If your data cannot be made publicly available for ethical or legal reasons (e.g., public availability would compromise patient privacy), please explain your reasons by return email and your exemption request will be escalated to the editor for approval. Your exemption request will be handled independently and will not hold up the peer review process, but will need to be resolved should your manuscript be accepted for publication. One of the Editorial team will then be in touch if there are any issues.

ANSWER

Additional information as tables and figures are uploaded to support our findings. However, we would not be able to publicly make the original dataset available. The reason is that: since this was carried during an internship, co-authors were able to supervise the first co-author on data collection, in such a way that the data serves as materials to other students. Thus, students are carrying their internship of the dataset; i.e. the dataset is still supporting current student research. In addition, **Although the whole dataset is being explored for secondary analysis, we uploaded the minimal dataset necessary to replicate the quantitative findings of this study, as well as the R code used, as a Supporting information file.**

Please review your reference list to ensure that it is complete and correct. If you have cited papers that have been retracted, please include the rationale for doing so in the manuscript text, or remove these references and replace them with relevant current references. Any changes to the reference list should be mentioned in the rebuttal letter that accompanies your revised manuscript. If you need to cite a retracted article, indicate the article’s retracted status in the References list and also include a citation and full reference for the retraction notice.

ANSWER

Thank you so much dear Editor for this interesting comment. We have revised the references and align them with the journal policy. We also added some recent reference in relation with Missed opportunities In the Country. One reference (number 10) stated as the first one in the domain that highlights the burden of MOV in the country; and we found quite interesting to cite it.

Reponses to Reviewers

PGPH-D-22-01589
Missed opportunities for vaccination and associated factors among children aged 0 to 23 months in Dschang health district, West region, Cameroon: a cross-sectional survey
PLOS Global Public Health

**Comments to the Author**

1. Does this manuscript meet PLOS Global Public Health’s [publication criteria](https://journals.plos.org/globalpublichealth/s/criteria-for-publication)? Is the manuscript technically sound, and do the data support the conclusions? The manuscript must describe methodologically and ethically rigorous research with conclusions that are appropriately drawn based on the data presented.

Reviewer #1: Partly

Reviewer #2: Yes

2. Has the statistical analysis been performed appropriately and rigorously?

Reviewer #1: Yes

Reviewer #2: Yes

ANSWER/

We are grateful for these positive comments from the reviewers. To increase the consistent of the results, we cross-checked the analysis, and clarify some missed points in the text. We re-ran the timeliness analysis and present the figure in the main text. We also used the occasion to improve the MOV analysis method by following the rigorous World health Organization methodology.

3. Have the authors made all data underlying the findings in their manuscript fully available (please refer to the Data Availability Statement at the start of the manuscript PDF file)?

The [PLOS Data policy](https://journals.plos.org/globalpublichealth/s/data-availability) requires authors to make all data underlying the findings described in their manuscript fully available without restriction, with rare exception. The data should be provided as part of the manuscript or its supporting information, or deposited to a public repository. For example, in addition to summary statistics, the data points behind means, medians and variance measures should be available. If there are restrictions on publicly sharing data—e.g. participant privacy or use of data from a third party—those must be specified.

Reviewer #1: No

ANSWER: the main dataset analyzed is used by a third party, reason why it can not be shared with the main text. However, we added the dataset used for the analysis, as well as the R code.

Reviewer #2: Yes

ANSWER

We are grateful, and thank the reviewer 2 for his comment that is aligned with our previous response to reviewer 1.

4. Is the manuscript presented in an intelligible fashion and written in standard English?

PLOS Global Public Health does not copyedit accepted manuscripts, so the language in submitted articles must be clear, correct, and unambiguous. Any typographical or grammatical errors should be corrected at revision, so please note any specific errors here.

Reviewer #1: Yes

Reviewer #2: Yes

ANSWER

We thank the editor for their comments. We used this opportunity to re-edit the manuscript and correct typo errors, and check for consistency within sentences. We do hope, the article is readable and understandable.

5. Review Comments to the Author

Please use the space provided to explain your answers to the questions above. You may also include additional comments for the author, including concerns about dual publication, research ethics, or publication ethics. (Please upload your review as an attachment if it exceeds 20,000 characters)

**#====================================**

**# SPECIFIC RESPONSES TO REVIEWER #1**

**#====================================**

Reviewer #1: Introduction
There are various typos in the introduction
"Indeed, it prevents nearly 75,000 children from suffering serious physical, mental or neurological disabilities [1]" this number doesn't seem correct . It is too low.
What is RR1 ?
The second last paragraph of introduction is more suited for discussion
**Answer: We thank the reviewer for the interesting comment and feedback. Of course, this seems too low regarding actual evidence: reason why the figure was deleted in the text.**

**RR1 stood for the first dose of measles administered at 9 months. This was replaced by MCV1, and defined in the text as the measles-rubella first dose.**

Methods
What is DHD?
what is meant by couples/children ? is there a better terminology that can be used ?
What is WHO quota sampling ?
What is CAP survey ?
**Answer: We thank the reviewer for the interesting comments that obviously drive our attention. The notation DHD stands for Dschang Health District. This notation has been revised and extended completely in the text. In some cases, we replaced it by “district” only.**

- **The term “couples/children” was replaced by the most suited expression. We meant children with their caregivers.**
- **“WHO quota sampling” refered to an existing literature that used the same sampling method recommended by the World Health Organization to estimate the number of health workers for the KAP study. To avoid misunderstanding, we simply reformulated that in the text, and mentioned that we set a quota of health workers, and divided that into the number of health facilities in the district.**
- **“CAP” study was a mistake; it stood for KAP; we have made the changes in the text, and provided the definition: knowledge, attitude/bahaviour and practice.**

Results

One decimal point should be enough
Please don't start sentences with numbers
Results are too long . some of the tables can be moved to appendix.
**Answer: We thank the reviewer for the interesting comments and apologize for all these insufficiencies. We went through the text and left numbers with one decimal point; sentences were revised as suggested, and we also put some tables as supplementary tables.**

Conclusion needs to be crisp and not more than 2-3 sentences

**Answer: We thank the reviewer for the remark. The conclusion has been shortened as suggested.**

**#====================================**

**# SPECIFIC RESPONSES TO REVIEWER #2**

**#====================================**

Reviewer #2: The manuscript numbering system was not done making review process difficult
Page 1
The title is topical as the EPI vaccination schedule in in developing countries and completion of RI is still a problem and the needs for more studies is appropriate

**ANSWER**

**We thank you for the comments, and added a little changes in the title.**

Abstract
....in the west region
MOV prevalence range from 69.7%(19/29) to 98.5%(330/335)
...74%asked parents
... strategies for remedying this includes
**Answer: All changes are done!**

Page 2
....remove expanded program on immunization after the ref
In Cameroon vaccination has been offered throughout the country
**Answer: We thank the reviewer for the attention made on the text. All changes are done!**

Table2: Types of vaccination...
Table 5: Sociodemographic information of health workers...
Table 9: Logistic regression of MOV among children...
**Answer: We thank the reviewer for the attention made on the text. All changes were made appropriately! After running the analysis, the logistic regression was no helpful, so the previous results were replaced by new ones, and we stated that more investigation is needed to explore factors that increase the risk of MOV.**

Discussion
Page 16
**Answer: We thank the reviewer pointing out this page for revision Indeed, we re-edited the text accordingly, and all changes are visible in the discussion.**

6. PLOS authors have the option to publish the peer review history of their article ([what does this mean?](https://journals.plos.org/globalpublichealth/s/editorial-and-peer-review-process#loc-peer-review-history)). If published, this will include your full peer review and any attached files.


**Do you want your identity to be public for this peer review?** If you choose “no”, your identity will remain anonymous but your review may still be made public.


For information about this choice, including consent withdrawal, please see our [Privacy Policy](https://www.plos.org/privacy-policy).

Reviewer #1: No

Reviewer #2: No

[NOTE: If reviewer comments were submitted as an attachment file, they will be attached to this email and accessible via the submission site. Please log into your account, locate the manuscript record, and check for the action link "View Attachments". If this link does not appear, there are no attachment files.]

While revising your submission, please upload your figure files to the Preflight Analysis and Conversion Engine (PACE) digital diagnostic tool, <https://pacev2.apexcovantage.com/>. PACE helps ensure that figures meet PLOS requirements. To use PACE, you must first register as a user. Registration is free. Then, login and navigate to the UPLOAD tab, where you will find detailed instructions on how to use the tool. If you encounter any issues or have any questions when using PACE, please email PLOS at [figures@plos.org](mailto:figures@plos.org). Please note that Supporting Information files do not need this step.
